# Provider perceptions of indications for red blood cell transfusion

**DOI:** 10.1111/trf.70045

**Published:** 2025-12-22

**Authors:** Aishwarya Katiki, Selwyn Rogers, Ryan Boudreau, David Meltzer, Micah Prochaska

**Affiliations:** ^1^ Pritzker School of Medicine University of Chicago Chicago Illinois USA; ^2^ Section of Trauma and Acute Care Surgery, Department of Surgery University of Chicago Chicago Illinois USA; ^3^ Section of Hospital Medicine, Department of Medicine University of Chicago Chicago Illinois USA

**Keywords:** RBC transfusion, transfusion practices (adult), transfusion practices (surgical)

## Abstract

**Background:**

Guidelines for red blood cell transfusion recommend incorporating patient factors and clinical context beyond hemoglobin (Hb) levels. However, limited data exist on which factors clinicians consider important. Understanding these decision‐making elements can clarify how guidelines are applied and inform future research. This study aimed to identify and prioritize factors that influence transfusion decisions among inpatient clinicians.

**Study Design and Methods:**

Inpatient clinicians who are high utilizers of transfusion were administered a survey and asked to rate the importance of 30 decision‐making factors using a 3‐point Likert scale (very, somewhat, not important). Additional questions addressed transfusion practices and anemia management using a 5‐point Likert scale (very much disagree to very much agree). Descriptive statistics were used to characterize study participants and survey responses, and regression models explored associations between responses and participant characteristics.

**Results:**

Of 95 eligible clinicians, 85 (89%) completed the survey. Only 7 of the 30 factors were rated as “very important” by more than 66% of respondents; 5 of these were Hb‐related. Importance assigned to other non‐Hb‐related factors varied. Most clinicians (85%) do believe that anemia can result in significant adverse consequences. Most clinicians further believe that restrictive transfusion is standard of care (88%) and optimal (68%), but also that transfusion decisions need to incorporate factors other than a patient's Hb level (84%) at the same time.

**Conclusion:**

Despite guidelines suggestions, there is a lack of consensus on what clinical factors beyond Hb clinicians believe are important in making transfusion decisions.

**Abbreviations**
TRALI: transfusion‐related acute lung injury
CI: confidence interval
EPIC: name of the electronic health record used at the University of Chicago
SD: standard deviation
TACO: transfusion‐associated circulatory overload

## 1 INTRODUCTION

Red blood cell (RBC) transfusion guidelines recommend restrictive transfusion practices and include a “good clinical practice statement,” encouraging transfusion decisions to be guided by patient factors and the clinical context, not just a single hemoglobin (Hb) value.^1^, ^2^, ^3^ This recommendation aligns with the long‐held idea that individual clinical and physiologic factors influence the need for and response to transfusion,^3^, ^4^, ^5^, ^6^ and clinicians recognize the need to individualize transfusion decisions based on factors other than patients' Hb level alone.^4^, ^7^, ^8^ For example, engaging in shared decision‐making about transfusion has been associated with improved outcomes, greater satisfaction, and overall cost savings.^9^ It is also recognized that transfusion trials have not been designed to evaluate all the factors that clinicians might consider when making a transfusion decision, including shared decision‐making,^5^ nor the optimal treatment of anemia.^10^ However, although guidelines support—and clinicians acknowledge—the need to individualize and base transfusion decisions on factors beyond Hb thresholds, there is an absence of data on whether this actually occurs in practice and which additional factors most influence these decisions.

Data on what factors clinicians consider important in making transfusion decisions in adults would be useful for understanding how transfusion guidelines are translated and applied in clinical practice. It would also be helpful for determining whether any identified variation in transfusion practice is clinically indicated, or inappropriate and potentially harmful.^11^ For instance, inappropriate use of RBC transfusion may cause severe complications such as TRALI, TACO, and hemolytic reactions.^12^ Last, it could be useful for identifying the important factors that drive transfusion practice but have not yet been adequately studied and could be the focus future transfusion research.^5^ Previous qualitative data collected in pediatric intensive care unit (PICU) providers does demonstrate that clinicians do consider a variety of non‐Hb‐related factors, including clinical and non‐clinical factors, in making transfusion decisions.^3^ However, hospitalized adults have different demographic, clinical, and physiologic characteristics, as well as logistic factors that might influence transfusion decisions. As a result, the purpose of this study was to identify such factors and the importance that clinicians give to such factors in influencing their transfusion decisions in hospitalized adult patients, as well as to understand how those factors relate to clinicians' general beliefs about transfusion practices and the treatment of anemia in hospitalized adults.

## 2 MATERIALS AND METHODS

### 2.1 Study design and participants

This was an observational study of clinicians (attending physicians and advanced practice providers) from the sections of Hospital Medicine and Trauma and Acute Care Surgery at the University of Chicago. These sections were chosen because the clinicians in both sections are high‐volume inpatient users of RBC transfusion, they represent different training backgrounds (medicine and surgery), and they care for patients with anemia who have different acute care hospital needs but are still subject to the same institutional transfusion recommendations. Any clinician from these clinical sections who provided direct patient care and can order RBC transfusion (attending physicians, fellows, and advanced practice providers) was eligible to participate, but non‐clinicians (i.e., PhD) were excluded. This study was approved by the University of Chicago Institutional Review Board.

### 2.2 Survey instrument

A survey to capture factors that might influence clinicians in making transfusion decisions and their beliefs about RBC transfusion practice and inpatient anemia treatment was developed using published literature, clinical experience, and feedback from institutional clinical and scientific leaders. The survey included demographic characteristics of the participating clinicians and asked respondents to rank using a 3‐point Likert scale (very important, somewhat important, not important) 30 different factors they might consider when deciding whether to transfuse a patient. The survey also included statements about standard transfusion practice and the inpatient treatment of anemia, and clinicians were asked to rank their level of agreement with these statements using a 5‐point Likert scale (very much agree, agree, neither agree nor disagree, disagree, very much disagree). The full survey can be found in Appendix S1.

### 2.3 Data collection

The study was administered in June and July of 2023 at in‐person section meetings. Any individual not present at the in‐person section meetings was provided an electronic version of the survey to complete.

### 2.4 Statistical analysis

Descriptive statistics were used to characterize the baseline demographic characteristics of study participants, the ranking of each factor influencing transfusion decisions, and answers to each survey question about transfusion and inpatient anemia management. Transfusion factors were ordered hierarchically, from highest to lowest, based on the number/percentage of “very important” responses each factor received by clinicians. Ordinal logistic regression models were used to test the association between provider characteristics and views on restrictive transfusion practice, and the association between the importance clinicians gave to “patient preference” (independent variable) and “shared decision‐making” (dependent variable) in transfusion decisions. Statistical tests were considered significant if the *p* < .05, and all statistical analysis was performed using STATA/MP 17.

Differences in the demographic characteristics and survey responses between hospitalist and trauma acute care providers were tested using Wilcoxon Rank‐Sum (continuous variables) or chi‐squared (categorical variables) tests. While there were a few differences in the baseline characteristics of hospitalists and trauma surgeons, there were no significant differences between these provider groups in their responses to the rankings of factors important in transfusion practice or the survey questions about transfusion practice and inpatient anemia management. As a result, we are reporting only the overall results from the full sample of participants.

## 3 RESULTS

### 3.1 Respondent characteristics

A total of 95 clinicians were eligible for participation, and of these 89% (85/95) completed the survey, although one respondent only completed the demographic section of the survey. The mean age of respondents was 39 years old, 49% (42/85) were female, 53% (45/85) were White, 76% (64/85) were attending physicians, and the mean number of years in practice was 8.3. Trauma surgery did have a higher percentage of Black (58% vs. 51%) and a lower percentage of Asian providers (13% vs. 36%) (*p* = .02), as well as fewer attending physicians (50% vs. 87%) and more advanced practice providers (42% vs. 10%) (*p* < .01) compared to hospital medicine (Table S1). The respondent characteristics for the overall sample are reported in Table 1.

**TABLE 1:** Baseline provider characteristics.

	*n* = 85
Age, mean (SD)	39 (7.0)
Sex, *n* (%)	
Female	42 (49)
Race, *n* (%)	
Asian	25 (29)
Black/African American	6 (7)
White	45 (53)
Other/prefer not to answer	9 (11)
Ethnicity, *n* (%)	
Not Hispanic or Latino	78 (92)
Hispanic or Latino	4 (5)
Prefer not to answer	3 (3)
Type of clinician, *n* (%)	
Attending physician	64 (76)
Resident/fellow physician	4 (5)
Advanced practice provider	16 (19)
Years in practice, mean (SD)	8.3 (5.7)

### 3.2 Importance of factors in transfusion decision‐making

**FIGURE 1:**
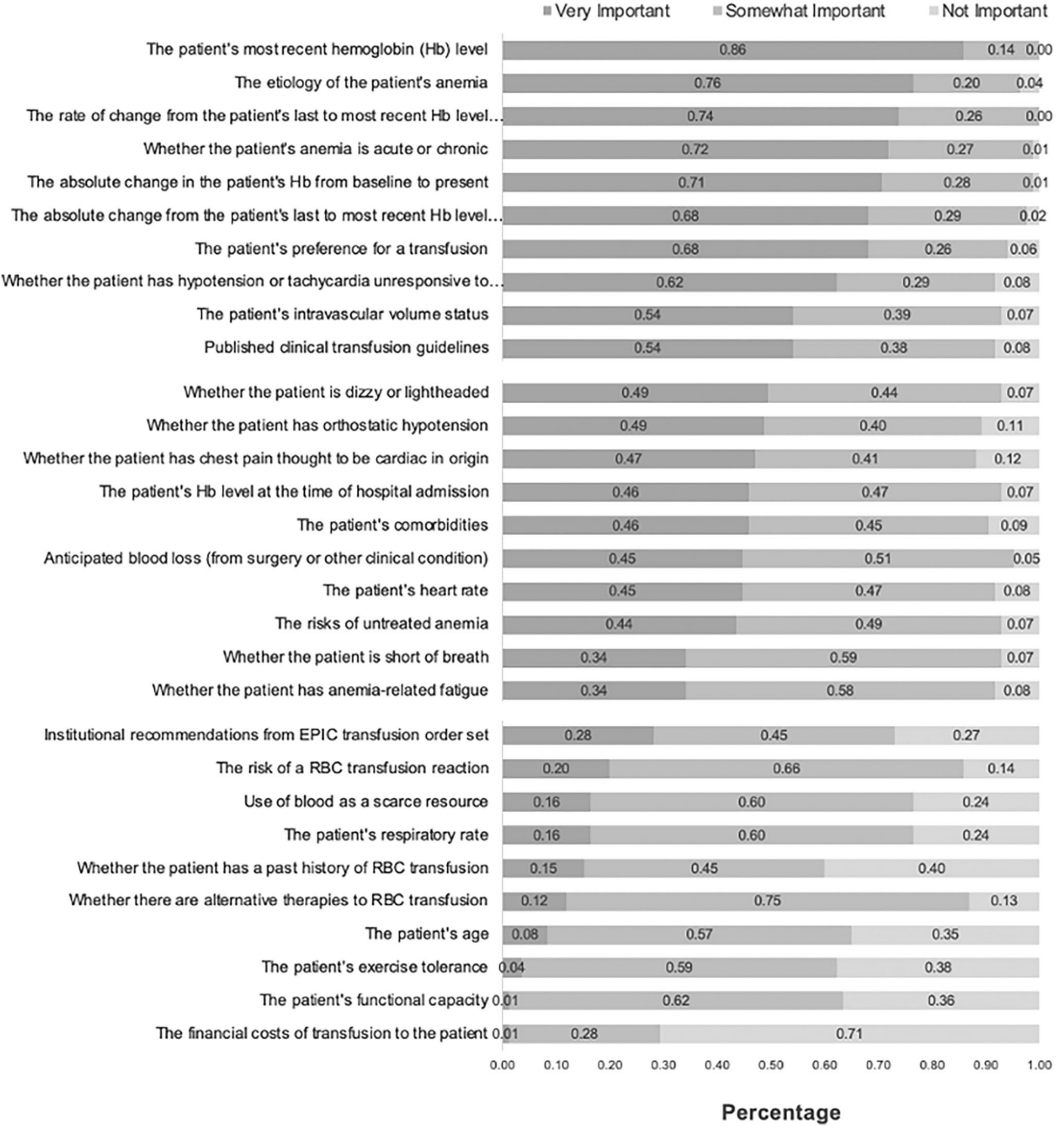
Importance of Factors in Transfusion Decision‐making.

The ranking of all factors ordered by providers' responses of “very important,” “somewhat important,” and “not important” are reported in Figure 1.

The top 10 factors ranked by the percent of clinicians labeling them “*very important*” included: The patient's most recent Hb level (86%); the etiology of the patient's anemia (76%), the rate of change from the patient's last to most recent Hb level during hospitalization (74%); whether the patient's anemia is acute or chronic (72%); the absolute change in the patient's Hb from baseline to present (71%); the absolute change from the patient's last to most recent Hb level during their hospitalization (68%); the patient's preference for a transfusion (68%); whether the patient has hypotension or tachycardia unresponsive to fluid challenge (62%); the patient's intravascular volume status (54%); and published clinical transfusion guidelines (54%). Of these top 10 factors only 7 had more than two‐thirds of clinician rate them as “very important,” and of those 7 factors 5 were related to the patient's Hb level.

The 10 least important factors ranked by the percent of doctors labeling them “*not important*” included: the financial costs of transfusion to the patient (71%); whether the patient has a past history of RBC transfusion (40%); the patient's exercise tolerance (38%); the patient's functional capacity (36%); the patient's age (35%); institutional recommendations from electronic health record (EHR) (i.e., EPIC) transfusion order set (27%); use of blood as a scarce resource (24%); the patient's respiratory rate (24%); the risk of a RBC transfusion reaction (14%); and whether there are alternatives to RBC transfusion (13%).

### 3.3 Clinicians view of restrictive transfusion practices and inpatient anemia management

**FIGURE 2:**
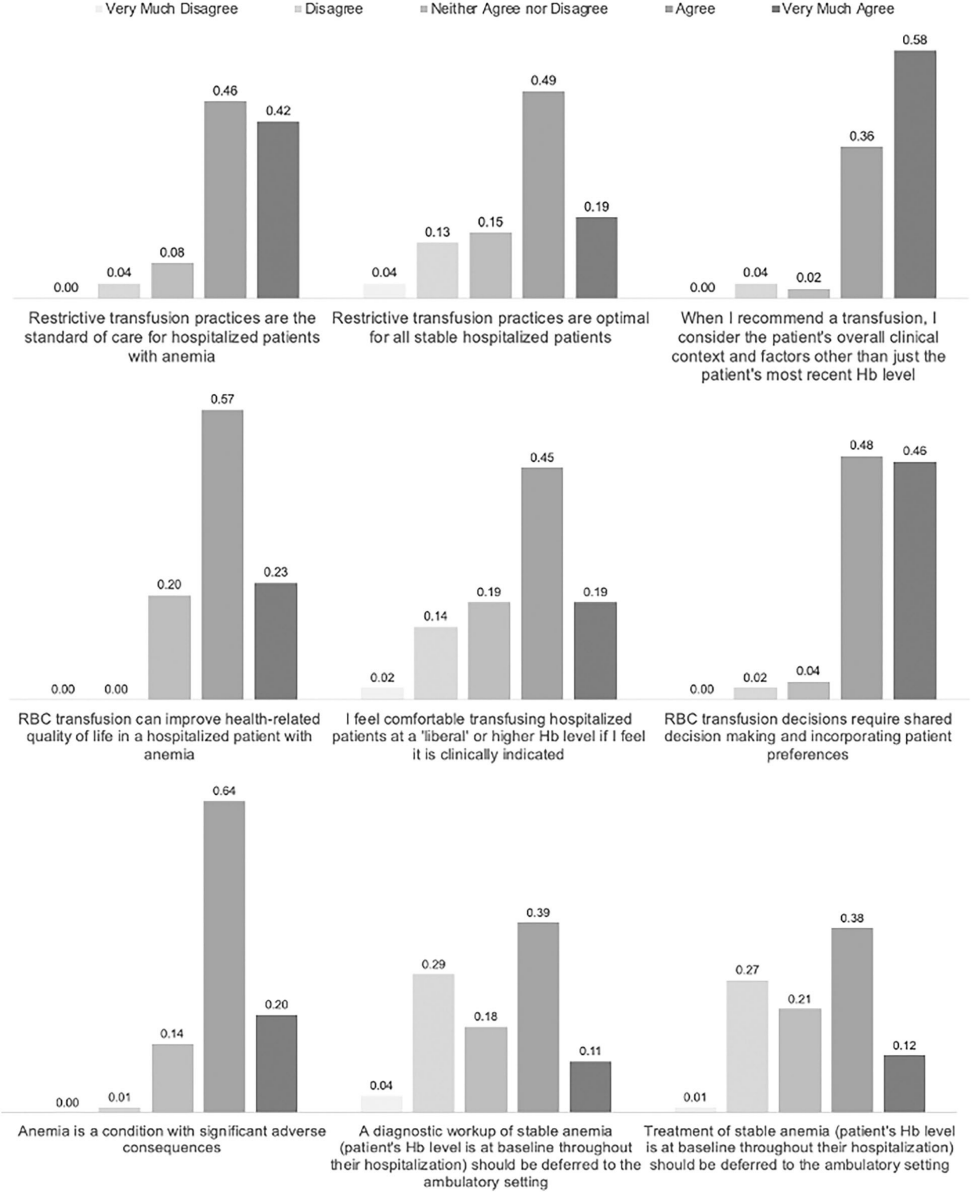
Clinician Views of Restrictive Transfusion Practice, Transfusion Decisions, and Inpatient Anemia Treatment.

Clinicians' views of restrictive transfusion practices, inpatient anemia treatment, and transfusion decision‐making are reported in Figure 2.

With respect to restrictive transfusion practices and guideline recommendations, 88% (74/84) of respondents very much agreed or agreed that restrictive transfusion is standard of care for hospitalized patients with anemia, and 89% (75/84) were in consensus that restrictive transfusion is characterized as less than 7 g/dL. However, only 68% (57/84) agreed or very much agreed that restrictive practice is an optimal treatment choice for all stable hospitalized patients with anemia, while 94% (79/84) very much agreed or agreed that they consider other patient factors and clinical context other than a patient's most recent Hb level when recommending a transfusion.

When asked about their general views of transfusion practices and inpatient anemia treatment, 80% (66/83) of respondents very much agreed or agreed that transfusion can improve health‐related quality of life, 64% (54/84) very much agreed or agreed that they would feel comfortable transfusing a patient at a liberal Hb threshold if it was clinically indicated, 85% (71/84) very much agreed or agreed that anemia is a condition that can result in significant adverse consequences, while 50% (42/84) very much agreed or agreed that the diagnostic work‐up and treatment of stable anemia should be deferred to the outpatient setting. Last, 94% (79/84) very much agreed or agreed that transfusion decisions require shared decision‐making with the patient.

### 3.4 Association between provider characteristics and restrictive transfusion practices

In regression models, older providers (OR 1.18, *p* < .01), men compared to women (OR 0.32, *p* = .04), and advanced practice providers compared to attending physicians (OR 70, *p* < .01) were more likely to report agreement with the belief that restrictive transfusion practices are standard of care. There were, however, no significant associations between provider characteristics and belief that restrictive transfusion practices are optimal or that clinical context matters in addition to Hb level when ordering transfusion for patients (Table 2).

**TABLE 2:** Association between provider characteristics and restrictive transfusion practices.

Provider characteristics	Restrictive transfusion practices are the standard of care for hospitalized patients	
	Odds ratio (95% CI)	*p*
Age	1.18 (1.06–1.34)	<.01
Sex (male = 1, female = 2)	0.32 (0.10–0.98)	.04
Race		
American Indian/Alaska Native	Referent	
Asian	0.05 (0.00–2.77)	.14
Black/African American	0.03 (0.00–2.63)	.13
White	0.03 (0.00–1.81)	.10
More than one race	0.01 (0.00–1.47)	.07
Prefer not to answer	0.31 (0.00–22.81)	.59
Attending	Referent	
Resident/fellow physician	4.1 (0.52–32.39)	.18
Advanced practice provider	70.3 (11.69–422.39)	.00
Years in practice	0.90 (0.79–1.04)	.15

### 3.5 Association between providers perception that patient preferences and shared decision‐making matter in transfusion decisions

Last, clinicians who ranked a patient's preference for a transfusion as “very important” had nearly 7 times higher odds of agreeing that transfusion decisions require shared decision‐making (OR 6.7, *p* = .06) (Table S2). This effect was not significant when adjusting for the age, sex, race, clinician type, and years in practice of a provider.

## 4 DISCUSSION

In this study of factors that might influence providers in making transfusion decisions, no single factor(s) were unanimously reported as very significant by providers. There were only seven factors that more than 67% of providers ranked as “very important,” with five of these seven being related to a patient's Hb level. Despite this, 94% of providers very much agreed or agreed that they consider the overall clinical context of the patient and factors outside of their Hb level when making transfusion decisions. These results highlight the tension between clinicians' belief that clinical factors other than Hb level influence the need for and response to a transfusion,^4^, ^5^ and the fact restrictive transfusion policies have emphasized Hb level alone as the primary relevant factor in making transfusion decisions. Interestingly, the RBC clinical transfusion guidelines suggest several clinical and physiologic factors that might influence transfusion (i.e., orthostatic hypotension, chest pain, and heart rate) as part of their good clinical practice statement,^1^, ^2^ but these factors had lower importance for clinicians in making transfusion decisions compared to Hb‐related factors. These clinical and physiologic factors are, however, critical for assessing a patient's volume status and the symptoms and burden a patient is experiencing from their anemia. This is particularly true in cases of high‐volume/acute hemorrhage where Hb is a less reliable marker in comparison to vitals due to fluid shifts (hemodilution, hemoconcentration, etc.). However, our data suggests that traditional factors may not be as relevant as previously thought and therefore underscores the need for further research into how transfusion decisions are truly made in clinical practice. There is a lack of literature investigating the actual factors that providers rely on in real‐world transfusion decision‐making, and this study aims to help fill this gap.

Our results also demonstrate that most providers agree that restrictive transfusion practices are not just standard of care in hospitalized adults, but that they are the optimal treatment approach to the treatment of anemia. Interestingly, we found that despite most providers believing that anemia is a significant condition with adverse consequences, only 50% of providers think a full diagnostic work‐up should be done in the hospital. This may be an inadvertent response to restrictive transfusion policies that emphasize the safety of transfusing only when a patient's Hb <7 g/dL, and the belief that therefore moderate anemia (Hb >7 g/dL) from any etiology in general is not significant enough to be worked up and/or treated during hospitalization. It is, however, problematic because patients have variable follow‐up appointments based on their admission etiology, length of stay, and access to outpatient care for working up, correcting, and managing their anemia. It also may explain why emerging data have demonstrated a high rate of iron deficiency anemia that can go untreated for years even when a patient interacts with the healthcare system.^13^

Our findings also suggest that additional studies and/or trials are needed to better understand whether such factors (i.e., clinical, physiological, external, etc.) can and should be used by providers to influence transfusion decisions, and their effect on important patient outcomes. Future work could include prospective studies that evaluate the impact of integrating these factors more explicitly into transfusion‐making algorithms. Integration of clinician decision support tools within the EHR, such as transfusion order sets, mandated dot phrases requiring documentation of shared decision‐making, or prompts recording which specific factors were considered during decision‐making, could help reduce variation in transfusion practice. Further studies incorporating the evaluation of these tools can help ensure providers are documenting not only Hb thresholds but also other relevant comorbidities as part of their procedure justification. These studies could evaluate further whether objectively using these factors in decision‐making can lead to improved patient outcomes, such as reduced transfusion‐related complications.

Our study has limitations. This was a prospective observational study of clinicians from a single academic medical center, and the outcomes may not be readily generalizable to other institutions. Additionally, our study captured providers' perceptions and beliefs about transfusion, but we do not have the data to link those perceptions and beliefs to the actual transfusion practices of the providers. Although we had an excellent response rate in two different groups of high transfusion utilizers, transfusion practices could vary significantly by other provider types. Additionally, our survey may have excluded important factors relevant in making transfusion decisions. Last, since questions asked in our survey utilized a Likert Scale response, which may have missed nuanced variation in providers' perceptions of the importance of these different factors.

## 5 CONCLUSION

Despite guideline recommendations to incorporate factors other than a patient's Hb level into transfusion decision‐making, Hb remains the predominant factor guiding transfusion practice. Moreover, there is no clear consensus among providers regarding which additional objective clinical and physiological factors should be considered when ordering a RBC transfusion.

## CONFLICT OF INTEREST STATEMENT

The authors have disclosed no conflicts of interest.

## Supporting information


**Table S1.** Baseline provider characteristics by provider type.
**Table S2**. Association between providers perception that patient preferences and shared decision‐making matter in transfusion decisions.

Appendix S1.

## References

[1] Carson JL , Stanworth SJ , Guyatt G , Valentine S , Dennis J , Bakhtary S , et al. Red blood cell transfusion: 2023 AABB international guidelines. JAMA. 2023;330(19):1892–1902. 10.1001/jama.2023.12914 37824153

[2] Carson JL , Guyatt G , Heddle NM , Grossman BJ , Cohn CS , Fung MK , et al. Clinical practice guidelines from the AABB: red blood cell transfusion thresholds and storage. JAMA. 2016;316(19):2025–2035. 10.1001/jama.2016.9185 27732721

[3] Steffen KM , Spinella PC , Holdsworth LM , Ford M , Lee GM , Asch SM , et al. Factors influencing pediatric transfusion: a complex decision impacting quality of care. Transfusion. 2023;63(6):1151–1160. 10.1111/trf.17364 37078686

[4] Klein HG , Flegel WA , Natanson C . Red cell transfusion: precision versus imprecision medicine. JAMA. 2015;314(15):1557–1558. 10.1001/jama.2015.10890 26355383 PMC5551903

[5] Natanson C , Applefeld WN , Klein HG . Hemoglobin‐based transfusion strategies for cardiovascular and other diseases: restrictive, liberal, or neither? Blood. 2024;144(20):2075–2082. 10.1182/blood.2024025927 39293024 PMC11600050

[6] Roubinian NH , Plimier C , Woo JP , Lee C , Bruhn R , Liu VX , et al. Effect of donor, component, and recipient characteristics on hemoglobin increments following red blood cell transfusion. Blood. 2019;134:1003–1013. 31350268 10.1182/blood.2019000773 PMC6764268

[7] Vincent JL . Which carries the biggest risk: anaemia or blood transfusion? Transfus Clin Biol. 2015;22(3):148–150. 10.1016/j.tracli.2015.05.001 26070458

[8] Sakr Y , Vincent JL . Should red cell transfusion be individualized? Yes. Intensive Care Med. 2015;41(11):1973–1976. 10.1007/s00134-015-3950-7 26149304

[9] Friedman M , Bizargity P , Gilmore S , Friedman A . Patient inclusion in transfusion medicine: current perspectives. Int J Clin Transfus Med. 2015;3:7–16. 10.2147/ijctm.s60919

[10] Shander A , Goodnough LT . From tolerating anemia to treating anemia. Ann Intern Med. 2019;170(2):125–126. 10.7326/M18-3145 30557445

[11] Willems SA , Kranenburg FJ , le Cessie S , Marang‐ van de Mheen PJ , Kesecioglu J , van der Bom JG , et al. Variation in red cell transfusion practice in the intensive care unit – an international survey. J Crit Care. 2020;55:140–144. 10.1016/j.jcrc.2019.10.003 31715532

[12] Shander A , Gross I , Hill S , Javidroozi M , Sledge S . A new perspective on best transfusion practices. Blood Transfus. 2013;11(2):193–202. 10.2450/2012.0195-12 23399354 PMC3626470

[13] Cogan JC , Meyer J , Jiang Z , Sholzberg M . Iron deficiency resolution and time to resolution in an American health system. Blood Adv. 2024;8(23):6029–6034. 10.1182/bloodadvances.2024013197 39145727 PMC11635663

