# Supplementary material for: Provider perceptions of indications for red blood cell transfusion
Source: Transfusion. 2025 Dec 22;66(2):326–33. doi: 10.1111/trf.70045 (PMC12902728; doi:10.1111/trf.70045)
Supplement: Supplementary file 1 — Table S1. Baseline provider characteristics by provider type. Table S2. Association between providers perception that patient preferences and shared decision‐making matter in transfusion decisions. [file TRF-66-326-s002.docx]

| ***Supplemental Table 1.*** *Baseline Provider Characteristics by Provider Type* | | | | |
| --- | --- | --- | --- | --- |
| **Characteristic** | **Overall**  (n = 85) | **Hospital Medicine**  (n = 61) | **Trauma Surgery**  (n= 24) | **p-value*** |
| **Age**,***** Mean (SD) | 39 (7.0) | 38 (6.5) | 41 (8.1) | 0.15 |
| **Sex**, n (%) |  |  |  | 0.30 |
| Female | 42 (49) | 28 (46) | 14 (58) |  |
| **Race**, n (%) |  |  |  | 0.02 |
| American Indian/Alaska Native | 1 (1) | 1 (2) | 0 (0) |  |
| Asian | 25 (29) | 22 (36) | 3 (13) |  |
| Black/African American | 6 (7) | 1 (2) | 5 (21) |  |
| White | 45 (53) | 31 (51) | 14 (58) |  |
| More than one race | 3 (4) | 2 (3) | 1 (4) |  |
| Prefer not to answer | 5 (6) | 4 (7) | 1 (4) |  |
| **Ethnicity**, n (%) |  |  |  | 0.53 |
| Not Hispanic or Latino | 78 (92) | 55 (90) | 23 (96) |  |
| Hispanic or Latino | 4 (5) | 3 (5) | 1 (4) |  |
| Prefer not to answer | 3 (4) | 3 (5) | 0 (0) |  |
| **Type of Clinician**,****** n (%) |  |  |  | <0.01 |
| Attending Physician | 64 (76) | 52 (87) | 12 (50) |  |
| Resident/Fellow Physician | 4 (5) | 2 (3) | 2 (8) |  |
| Advanced Practice Provider | 16 (19) | 6 (10) | 10 (42) |  |
| **Years in Practice,** Mean (SD) | 8.3 (5.7) | 8.0 (5.3) | 9.1 (6.89) | 0.65 |
| *P-value is for Wilcoxon Rank-Sum test for continuous variables and Chi-squared for categorical variables | | | | |

***Supplemental Table 2.*** *Association Between Providers Perception that Patient Preferences and Shared Decision-Making Matter in Transfusion Decisions*

| **How Important is a Patient’s Preference for Transfusion** | **Transfusion Decisions Require Shared Decision Making** | |
| --- | --- | --- |
|  | *Odds Ratio (95% CI)* | *p* |
| *Not Important* | *Referent* |  |
| *Somewhat Important* | *0.85 (0.10-7.11)* | *0.88* |
| *Very Important* | *6.7 (0.91-49.82)* | *0.06* |
| *Age* | *0.9 (0.85-1.06)* | *0.34* |
| *Sex (male =1, female =2)* | *1.7 (0.55-5.53)* | *0.35* |
| *Race (all subsets)* | *0 (0)* | *0.99* |
| *Attending* | *Referent* |  |
| *Resident/Fellow Physician* | *3.3 (0.21-49.94)* | *0.40* |
| *Advanced Practice Provider* | *0.7 (0.15-3.33)* | *0.66* |
| *Years in Practice* | *1 (0.89-1.18)* | *0.71* |
