# Supplementary material for: Provider perceptions of indications for red blood cell transfusion
Source: Transfusion. 2025 Dec 22;66(2):326–33. doi: 10.1111/trf.70045 (PMC12902728; doi:10.1111/trf.70045)
Supplement: Supplementary file 2 — Appendix S1. [file TRF-66-326-s001.pdf]

Red Blood Cell (RBC) Transfusion in Patients with Anemia

We are conducting a research study at the University of Chicago regarding provider perceptions of red blood cell (RBC) transfusion indications. The purpose of this research is to collect data on how providers prioritize different factors when considering transfusion, and better gauge their understanding of current clinical transfusion guidelines. Research participation is voluntary, does not involve any risk beyond that of everyday life, and will not influence employment or career advancement. Participation should take about 10 minutes.

If you have any questions or concerns about the study, you can contact the investigators at mprochas@bsd.uchicago.edu (Dr. Micah Prochaska) or aishwarya.katiki@uchicagomedicine.org (Aishwarya Katiki).

Thank you.

Please give the first 2 letters of your mother's first name + the last 4 digits of your cell phone number + the first 2 letters of your father's first name

[ex. um9293bh]

____

---

1. What is your age?

____

---

2. What is your sex?

○ Male
○ Female

---

3. What is your race?

○ American Indian/Alaska Native
○ Asian
○ Native Hawaiian or Other Pacific Islander
○ Black/African American
○ White
○ More than one race
○ Prefer not to answer

---

4. What is your ethnicity?

○ Not Hispanic or Latino
○ Hispanic or Latino
○ Prefer not to answer

---

5. What type of Clinician are you?

○ Attending Physician
○ Resident/Fellow Physician
○ Advanced Practice Provider

---

6. What is your area of practice?

○ Medicine
○ Surgery

---

7. How many years have you been in practice?

____

8. When considering a RBC transfusion for a hospitalized patient, how important are the following in your decision:

	Very Important	Somewhat Important	Not Important
The patient's most recent hemoglobin (Hb) level	○	○	○
The patient's Hb level at the time of hospital admission	○	○	○
The absolute change in the patient's Hb from baseline to present	○	○	○
The absolute change from the patient's last to most recent Hb level during their hospitalization	○	○	○
The rate of change from the patient's last to most recent Hb level during hospitalization	○	○	○
The patient's age	○	○	○
The patient's heart rate	○	○	○
The patient's respiratory rate	○	○	○
The patient's intravascular volume status	○	○	○
The patient's exercise tolerance	○	○	○
The patient's functional capacity	○	○	○
The patient's comorbidities	○	○	○
The patient's preference for a transfusion	○	○	○
The etiology of the patient's anemia	○	○	○
Whether the patient's anemia is acute or chronic	○	○	○
Whether the patient has orthostatic hypotension	○	○	○
Whether the patient is short of breath	○	○	○
Whether the patient is dizzy or lightheaded	○	○	○
Whether the patient has anemia-related fatigue	○	○	○
Whether the patient has a past history of RBC transfusion	○	○	○
Whether the patient has chest pain thought to be cardiac in origin	○	○	○

Whether the patient has hypotension or tachycardia unresponsive to fluid challenge	◯	◯	◯
Published clinical transfusion guidelines	◯	◯	◯
Institutional recommendations from EPIC transfusion order set	◯	◯	◯
Whether there are alternative therapies to RBC transfusion	◯	◯	◯
Use of blood as a scarce resource	◯	◯	◯
The financial costs of transfusion to the patient	◯	◯	◯
The risk of a RBC transfusion reaction	◯	◯	◯
The risks of untreated anemia	◯	◯	◯
Anticipated blood loss (from surgery or other clinical condition)	◯	◯	◯

---

9. Restrictive RBC transfusion practice is best defined as transfusion when a hospitalized patient's Hb (g/dL) is:

◯ >= 10.0
◯ 9.9 - 9.0
◯ 8.9 - 8.0
◯ 7.9 - 7.0
◯ < 7.0
◯ I do not know

---

10. If a patient said they were fatigued, what is the highest Hb level (g/dL) at which would you recommend a transfusion?

◯ >= 10.0
◯ 9.9 - 9.5
◯ 9.4 - 9.0
◯ 8.9 - 8.5
◯ 8.4 - 8.0
◯ 7.9 - 7.5
◯ 7.4 - 7.0
◯ < 7.0

---

11. If a patient said they were NOT fatigued, what is the highest Hb level at which would you recommend a transfusion?

◯ >= 10.0
◯ 9.9 - 9.5
◯ 9.4 - 9.0
◯ 8.9 - 8.5
◯ 8.4 - 8.0
◯ 7.9 - 7.5
◯ 7.4 - 7.0
◯ < 7.0

---

12. A patient presents with a GI bleed, is hemodynamically stable, and has an initial Hb level of 10.5 g/dL in the emergency room. If their Hb were to drop, at what level would you recommend an RBC transfusion?

◯ >= 10.0
◯ 9.9 - 9.5
◯ 9.4 - 9.0
◯ 8.9 - 8.5
◯ 8.4 - 8.0
◯ 7.9 - 7.5
◯ 7.4 - 7.0
◯ < 7.0

13. A patient presents without a GI bleed, is hemodynamically stable, and has an initial Hb level of 10.5 g/dL in the emergency room. If their Hb were to drop, at what level would you recommend an RBC transfusion?

- ◯ >= 10.0
- ◯ 9.9 - 9.5
- ◯ 9.4 - 9.0
- ◯ 8.9 - 8.5
- ◯ 8.4 - 8.0
- ◯ 7.9 - 7.5
- ◯ 7.4 - 7.0
- ◯ < 7.0

14. Please state how much you agree or disagree with the following statements:

	Very Much Agree	Agree	Neither Agree nor Disagree	Disagree	Very Much Disagree
Restrictive transfusion practices are the standard of care for hospitalized patients with anemia	○	○	○	○	○
Restrictive transfusion practices are optimal for all stable hospitalized patients	○	○	○	○	○
When I recommend a transfusion, I consider the patient's overall clinical context and factors other than just the patient's most recent Hb level	○	○	○	○	○
RBC transfusion can improve health-related quality of life in a hospitalized patient with anemia	○	○	○	○	○
I feel comfortable transfusing hospitalized patients at a "liberal" or higher Hb level if I feel it is clinically indicated	○	○	○	○	○
Anemia is a condition with significant adverse consequences	○	○	○	○	○
A diagnostic workup of stable anemia (patient's Hb level is at baseline throughout their hospitalization) should be deferred to the ambulatory setting	○	○	○	○	○
Treatment of stable anemia (patient's Hb level is at baseline throughout their hospitalization) should be deferred to the ambulatory setting	○	○	○	○	○
Single unit RBC transfusions are preferred over multiple unit transfusions in all clinical circumsntances	○	○	○	○	○
Variation in RBC transfusion practice across providers results in poor quality of care for patients	○	○	○	○	○

Variation in RBC transfusion practice across providers results in increased medical costs for patients	○	○	○	○	○
A stable hospitalized patient with iron deficient anemia should receive a transfusion if their Hb is less than 7g/dL	○	○	○	○	○
I would consider recommending transfusion in a stable hospitalized patient with iron deficient anemia with a Hb above 7g/dL	○	○	○	○	○
Oral iron is preferred to intravenous iron replacement for hospitalized patients with iron deficient anemia	○	○	○	○	○
RBC transfusion decisions require shared decision making and incorporating patient preferences	○	○	○	○	○

15. Please select the approximate risk from a unit of transfusion for a:

	< 1%	> 1%	I do not know
Febrile reaction	○	○	○
Transfusion-associated ciculatory overload	○	○	○
Allergic Reaction	○	○	○
Transfusion related acute lung injury (TRALI)	○	○	○
Transfusion transmitted HIV, Hepatitis B, or Hepatitis C	○	○	○
Fatal Hemolysis	○	○	○
